# Initial assessment and treatment of refugees in the Mediterranean Sea (a secondary data analysis concerning the initial assessment and treatment of 2656 refugees rescued from distress at sea in support of the EUNAVFOR MED relief mission of the EU)

**DOI:** 10.1186/s13049-016-0270-z

**Published:** 2016-05-20

**Authors:** M. Kulla, F. Josse, M. Stierholz, B. Hossfeld, L. Lampl, M. Helm

**Affiliations:** Department of Anaesthesiology and Intensive Care Medicine, German Armed Forces Hospital Ulm, Section Emergency Medicine, Oberer Eselsberg 40, 89081 Ulm, Germany; Frigate Schleswig-Holstein, Ship Medical Officer, Endraßstrasse, 26382 Wilhelmshaven, Germany

**Keywords:** Refugee, Asylum seeker, Mediterranean Sea, Triage, Initial assessment, Initial treatment, German Armed Forces

## Abstract

**Background:**

As a part of the European Union Naval Force – Mediterranean Operation Sophia (EUNAVFOR Med), the Federal Republic of Germany is contributing to avoid further loss of lives at sea by supplying two naval vessels. In the study presented here we analyse the medical requirements of such rescue missions, as well as the potential benefits of various additional monitoring devices in identifying sick/injured refugees within the primary onboard medical assessment process.

**Methods:**

Retrospective analysis of the data collected between May – September 2015 from a German Naval Force frigate. Initial data collection focused on the primary medical assessment and treatment process of refugees rescued from distress at sea. Descriptive statistics, uni- and multivariate analysis were performed. The study has received a positive vote from the Ethics Commission of the University of Ulm, Germany (request no. 284/15) and has been registered in the German Register of Clinical Studies (no. DRKS00009535).

**Results:**

A total of 2656 refugees had been rescued. 16.9 % of them were classified as “medical treatment required” within the initial onboard medical assessment process. In addition to the clinical assessment by an emergency physician, pulse rate (PR), core body temperature (CBT) and oxygen saturation (SpO_2_) were evaluated. Sick/injured refugees displayed a statistically significant higher PR (114/min vs. 107/min; *p* < .001) and CBT (37.1 °C vs. 36.7 °C; *p* < .001). There was no statistically significant difference in SpO_2_-values. The same results were found for the subgroup of patients classified as “treatment at emergency hospital required”. However, a much larger difference of the mean PR and CBT (35/min resp. 1.8 °C) was found when examining the subgroups of the corresponding refugee boats. A cut-off value of clinical importance could not be found. Predominant diagnoses have been dermatological diseases (55.4), followed by internal diseases (27.7) and trauma (12.1 %). None of the refugees classified as “healthy” within the primary medical assessment process changed to “medical treatment required” during further observation.

**Conclusions:**

The initial medical assessment by an emergency physician has proved successful. PR, CBT and SpO_2_ didn’t have any clinical impact to improve the identification of sick/injured refugees within the primary onboard assessment process.

**Electronic supplementary material:**

The online version of this article (doi:10.1186/s13049-016-0270-z) contains supplementary material, which is available to authorized users.

## Background

Since mid-2015, the tabloid press has been reporting almost daily on the refugee crisis that has been ongoing in the Mediterranean for several years. The currently known migration routes across the Mediterranean go from Libya to Sicily, Malta and Greece. In addition to various non-governmental organisations such as “Médecins Sans Frontières”, countries bordering the Mediterranean are making efforts to at least improve the refugees’ chances of survival. This flow of migrants from Africa and the Middle East towards Europe has led to the need for an extensive European Union humanitarian support mission (EUNAVFOR MED). Germany is participating in this mission with two Navy vessels [1].

The key medical challenges of this mission have included the urgent initial assessment and treatment of hundreds of rescued persons in distress at sea per refugee boat and the prevailing climate conditions. In the light of these demands, the medical crew on board the deployed frigate has been enlarged by additional medical personnel. Besides his normal team, consisting of three paramedics, the ship’s medical officer has been supported by a

- ▪ physician (emergency physician/anaesthesiologist^1^) and a
- ▪ specialist nurse for anaesthesia and intensive care medicine.

### The situation regarding the refugees

Many of the refugees have undergone long mental and physical ordeals that have forced them to leave their country [2]. They then face the additional agony of a long and unsafe flight. Before they can get onto one of the small refugee boats, they often have to wait for days or weeks in wretched and cramped conditions near the coast. As a result, the refugees are already weak and have numerous injuries/illnesses when they embark across the Mediterranean. This alone explains why up to 60 % of them are in a worrying general medical condition [3].

### The rescue procedure for people in distress at Sea

**Fig. 1:**
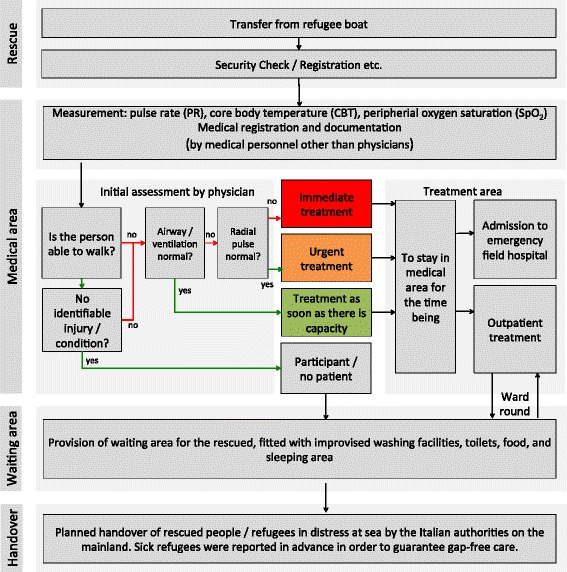
Chart of patient flow and medical care provided to refugees rescued from distress at. The focus of the chart is on initial assessment and medical care: First measurement of CBT, SpO_2_ and PR by paramedics, than a rough inspection and examination of the refugees by an experienced emergency physician (general condition, nutritional condition, exposed skin, eyes, hands, parts of the body the patients chose to show, and palpation of the peripheral pulse of the radial artery [31]) (Abbr.: SpO_2_ = peripheral oxygen saturation; CBT = core body temperature PR = pulse rate) Green arrows indicate a positive decision; red arrows a negative)

The rescue procedure – from the initial assessment to the handover on the European mainland in Italy is illustrated in Fig. 1.

### Personal protection measures and working conditions

The risk of rescue personnel becoming infected by these people in distress at sea is fundamentally rated as “medium to high” [2–5]. Measures have been taken to protect the involved ship’s crew against potential infections [4–6] when handling the people in distress at sea:

1. A double door system has been installed between the crew area of the ship and the area in which the rescued people are accommodated.
2. Personnel dealing with the rescued people are required to wear personal protective equipment (PPE) comprising disposable gloves, protective goggles, a protective respiratory mask (type FFP 3), and full-body protective suits (type III category 5/6) [6].

### Medical examination and treatment options

When taken on board, each person was provided with a numbered wristband and, after being searched for dangerous objects, has underwent an initial medical assessment. Communication was barely been possible due to the language barrier. Non-verbal communication was also very limited because of the PPE.

Rescued people identified as sick/injured (cf. Fig. 1) were taken to a separate area for further treatment. Those classified as healthy received further support in the waiting area. All treated patients were visited again the following day. Most of the people were handed over in Italy the following day – within 24 h, or 36 h at the latest.

### Questions

- ▪ Is the medical infrastructure suitable for a rescue mission of this nature?
- ▪ Are the technical examinations carried out as part of the initial assessment (PR, CBT and SpO_2_) suitable for identifying sick or injured patients?

## Material and methods

This study is a secondary data analysis [7]. Raw data was collected during the initial assessment and medical treatment of people rescued from distress at sea on a German Navy (Bundeswehr) frigate between May 2015 and September 2015. The STROBE [8] recommendations and the recommendations for performing a secondary data analysis [9] as well as observational studies [10] have been applied. The study has received a positive vote from the Ethics Commission of the University of Ulm, Germany (request no. 284/15) and has been registered in the German Register of Clinical Studies (no. DRKS00009535) [11]. The study was performed in compliance with the Helsinki Declaration (October 2013). Need for written consent was waived, as the study collected anonymised data.

### Inclusion and exclusion criteria

All people rescued from distress at sea by the frigate during the study period were included in the study. There were no exclusion criteria.

### Variables examined

An excerpt of the variables examined is available as Additional file 1. The term “sick” is used for a rescued person/refugee in distress at sea diagnosed as sick/injured while on board the frigate. “Sick” patients needed medical help by a physician. The term “sick” is used to mean the same as “injured” or “patient”. In case of treatment in the provisional field hospital on board of the frigate they were classified as “admission in the emergency field hospital”. All other rescued persons are defined as “participants”.

Because of a planned team rotation, two emergency physicians with equal qualification carried out the described triage by physician. All other personnel involved in the study stayed the whole time in the mission.

### Data processing and analysis

All the data was documented in parallel with the patients’ treatment by means of a study database. Healthy people (= participants) did not undergo further examination. For documentation purposes, a triage tag was used for each sick/injured patient. The medical measures conducted were also specified in the study database. No further follow-on examination was performed for study purposes. To pass on the information collected, all the triage tags were handed over to the medical personnel on the Italian mainland (cf. Fig. 1). A secondary feedback about the treatment on the Italian mainland regarding confirmation of preliminary on board diagnosis or missed diagnoses was not possible in this study setting.

### Biometric methods

Firstly, a descriptive statistic was performed calculating percentages for nominal and ordinal variables. The median, the algebraic average, standard deviation (SD), and the 95 % confidence interval of the average value are calculated for constant variables. Before statistical test methods for univariate analysis were used, the normal distribution of continuous variables was checked (Shapiro-Wilk test). Two independent samples underwent conservative statistical tests using the Mann–Whitney-*U* test, and more than two independent samples were examined using the Kruskal-Wallis test. Categorical data was tested using the Pearsons Chi-Square Test. Continuous variables like CBT, PR and SpO_2_ were additionally classified/coded in normal findings vs. pathological findings (cf. Additional file 1). At least uni- and multivariate binary logistic regression analysis were performed to investigate the classified/coded vital sings’ association to the health status of the rescued person [12]. A p-value <5 % was rated significant. An exclusion on variable level was carried out [13] when values were not available. All analysis presentations and figures were created using PowerPoint for MacOS 2011 (Microsoft Corp., Redmond, USA) and IBM SPSS Statistics 23 (IBM, Armonk, USA).

## Results

### General

During the study period, 2656 people in distress at sea were rescued from ten refugee boats and taken to mainland Italy within 24 to 36 h. Between 84 and 540 people (median = 188.5) could be rescued per refugee boat (cf. Table 1). The people rescued from the individual refugee boats differed significantly both in terms of age structure and in terms of the proportion of sick/injured people per boat. No deaths were reported. 18 (3.4 %) of the 538 female adults rescued stated that they were pregnant and one woman gave birth to a healthy child on board the frigate.

**Table 1:** Summary of demographic data of all people/refugees rescued from distress at sea

	Overall refugee population
Number [n]	*n* = 2656
Male Gender	2048/2656 (77.1 %)
Age structure	
▪ Infant	19/2656 (0.7 %)
▪ Child	274/2656 (10.3 %)
▪ Adult	2351/2656 (88.5 %)
▪ Elderly	31/2656 (1.2 %)
Pregnant women	24/2656 (0.9 %)
Sick patients	448/2656 (16.9 %)
Admission to Emergency field hospital^a^	63/2056 (3.1 %)

Note: ^a^Admission to emergency field hospital was not recorded for refugee boats nos. 1 and 2

### Initial assessment and triage

In all, 16.9 % of the people rescued were identified during the assessment carried out by a physician as being in need of medical treatment. None of the patients was categorised as “immediate = red” or “urgent = orange” (cf. Fig. 1). Here, the data is 100 % complete.

With respect to the additional determination of vital signs by medical personnel other than physicians, the information were complete in the case of 72.1 % of those rescued. One noticeable feature is that the information were incomplete for virtually only four refugee boats. The survey rate for the remaining six refugee boats could be classified as complete.

The averages pulse rate were 108/min, those of core body temperature 36.8 °C, and those of peripheral oxygen saturation 97 %.

**Fig. 2:**
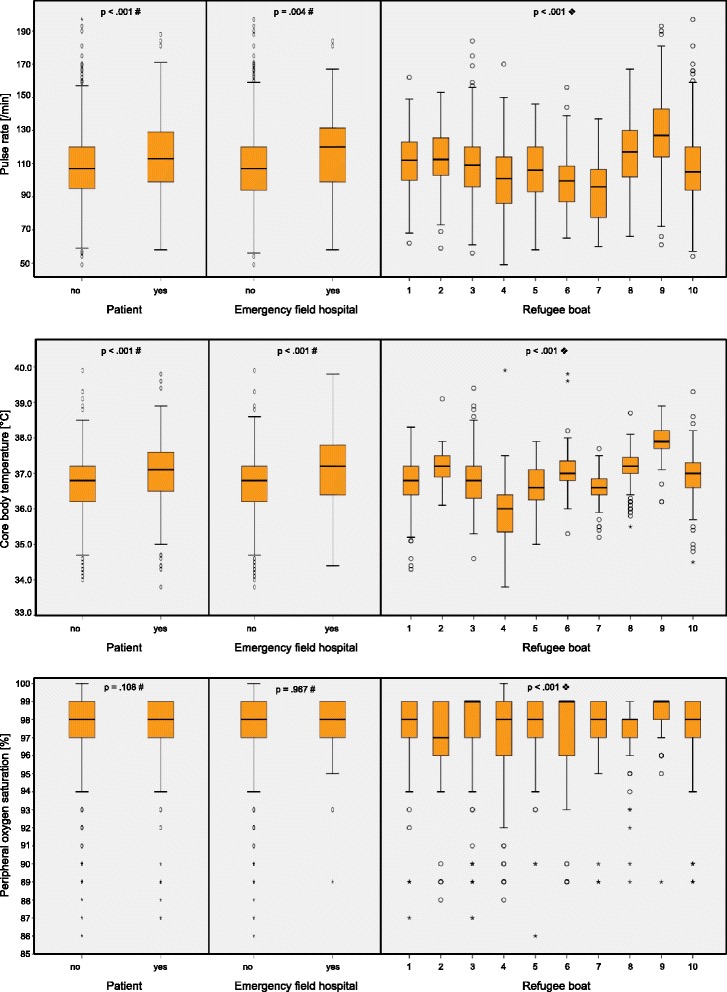
**a**-**c**: Chart showing PR (pulse rate - **a**), CBT (core body temperature -**b**) and SpO_2_ (peripheral oxygen saturation - **c**) as part of initial assessment by medical personnel other than physicians as grouped boxplots. Subgroup analysis of data, based on state of health (sick patient/healthy participant), treatment at emergency field hospital (yes/no) and refugee boat. Note: # Mann–Whitney-*U* Test . ❖ Kruskal-Wallis Test

Figure 2a-c show the vital parameters collected for different subgroups. The left-hand columns of Fig. 2a-c illustrate that healthy refugees had a significantly lower average pulse rate (PR_healthy_ = 107/min vs. PR_sick_ = 114/min *p* < .001). Core body temperature also differed considerably between healthy and sick people (CBT_healthy_ = 36.7 °C vs. CBT_sick_ = 37.1 °C *p* < .001). In contrast, the oxygen saturation did not allow any statistical conclusions to be drawn about the people’s state of health. Gender had no influence on CBT or SpO_2_, but it did affect average pulse rate (PR_men_ = 106/min vs. PR_women_ = 116/min *p* < .001).

Several binary logistic regression models were calculated in order to estimate if it is possible to predict whether a rescued person would be sick or injured (cf. Tables 2 and 3). None of them had a suitable sensitivity/specificity. The best achievable sensitivity was 54.0 % with a corresponding specificity of 81.4 % with the model, which has the refugees’ boat included.

**Table 2:** Absolute numbers and percentages of people rescued from distress at sea in the defined categories of vital signs (Categorised values of pulse rate (PR), core body temperature (CBT) and peripheral oxygen saturation (SpO_2_))

	Category							
PR [/min]	≤39	40-49	50-59	60-80	81-120	121-150	≥151	Missing data
Healthy participants	0 (0.0 %)	1 (0.1 %)	5 (0.3 %)	174 (9.0 %)	1027 (53.0 %)	344 (17.7 %)	31 (1.6 %)	717 (27.0 %)
Sick patients	0 (0.0 %)	0 (0.0 %)	1 (0.1 %)	32 (1.7 %)	200 (10.3 %)	101 (5.2 %)	23 (1.2 %)	
CBT [°C]		≤34.9	35.0-35.9	36.0-37.5	37.6-39.0	≥39.1		Missing data
Healthy participants		37 (1.7 %)	246 (11.1 %)	1384 (62.4 %)	160 (7.2 %)	3 (0.1 %)		439 (16.5 %)
Sick patients		7 (0.3 %)	30 (1.4 %)	247 (11.1 %)	100 (4.5 %)	3 (0.1 %)		
SpO_2_ [%]	≤84	85-89	90-95	96-100				Missing data
Healthy participants	6 (0.3 %)	33 (1.7 %)	76 (3.9 %)	1469 (75.7 %)				716 (27.0 %)
Sick patients	0 (0.0 %)	12 (0.6 %)	12 (0.6 %)	332 (17.1 %)

**Table 3:** Coefficients of the binary logistic regression model predicting whether a rescued person will be sick/injured. Three different models are calculated: In the left column the results of the univariate binary logistic model are demonstrated. The middle column shows the results of the multivariate binary logistic regression without including the information of the refugees boat. The right column includes all information in one model. (Abbr.: regression coefficient (R), p-Value (p), calculated Odds Ratio (OR) with its corresponding 95 % confidence interval (95 % CI)

Variable	Category	Univariate binary logistic regression model			Multivariate binary logistic regression model without refugee boat Nagelkerkes R^2^ = .09 Sensitivity: 29.7 % Specifity = 89.4 % (Cut-Off: .25)			Multivariate binary logistic regression with refugee boat included: Nagelkerkes R^2^ = .21 Sensitivity: 54.0 % Specifity: 81.4 % (Cut-Off: .25)		
		R	P	OR [95 % CI]	R	P	OR [95 % CI]	R	P	OR [95 % CI]
PR [/min]	≤39	not calculated ^a^			not calculated ^a^			not calculated ^a^		
	40-49	−21.20	1.000	not calculated ^a^	not calculated ^a^			not calculated ^a^		
	50-59	−1.61	0.14	0.20 [0.02-1.71]	−1.18	.310	0.31 [0.03 - 2.99]	−0.61	.617	0.54 [0.05-5.91]
	60-80	−1.69	< .001	0.18 [0.13-0.27]	−1.07	.004	0.34 [0.17 - 0.71]	−0.58	.146	0.56 [0.26-1.22]
	81-120	−1.64	< .001	0.20 [0.17-0.23]	−0.96	.002	0.38 [0.21 - 0.71]	−0.75	.027	0.47 [0.24-0.92]
	121-150	−1.23	< .001	0.29 [0.24-0.37]	−0.71	.025	0.49 [0.27 - 0.92]	−0.57	.101	0.57 [0.29-1.12]
	≥151	−0,30	.287	0.74 [0.43-1.27]	not included ^b^			not included ^b^		
CBT [°C]	≤34.9	−1.67	< .001	0.19 [0.08-0.42]	−1.18	.264	0.31 [0.04 - 2.43]	−0.47	.676	0.63 [0.07-5.57]
	35.0 – 35.9	−2.10	< .001	0.12 [0.08-0.18]	−1.58	.062	0.21 [0.04 -1.09]	−1.33	.145	0.26 [0.04-1.58]
	36.0 – 37.5	−1.72	< .001	0.18 [0.15-0.20]	−1.53	.065	0.22 [0.04 - 1.10]	−1.69	.056	0.18 [0.03-1.05]
	37.6 – 39.0	−0.47	< .001	0.63 [0.49-0.80]	−0.38	.653	0.69 [0.13 -3.54]	−1.18	.193	0.31 [0.05-1.81]
	≥39.1	0.00	1.000	1.00 [0.20-5.0]	not included ^b^			not included ^b^		
SpO2 [%]	≤84	−21.20	1.000	not calculated ^a^	not calculated ^a^			not calculated ^a^		
	85-89	−1.01	.003	0.36 [0.19-0,70]	1.22	.339	3.37 [0.28 - 40.88]	0.20	.886	1.22 [0.08-17.71]
	90-95	−1.85	< .001	0.16 [0.07-0.29]	0.28	.822	1.33 [0.11 - 15.91]	−0.64	.639	0.53 [0.04-7.55]
	96-100	−1.49	< .001	0.23 [0.20-0.26]	0.52	.669	1.68 [0.15-18.39]	−0.54	.677	0.58 [0.05-7.52]
Age category	Infant	−2.83	.006	0.06 [0.01-0.44]	−0.21	.880	0.81 [0.05 -12.21]	−0.70	.624	0.50 [0.03-8.10]
	Child	−1.80	< .001	0.17 [0.12-0.23]	−0.14	.873	0.87 [0.16 -4.72]	−0.35	.695	0.70 [0.12-4.08]
	Adult	−1.58	< .001	0.21 [0.19-0.23]	−0.39	.638	0.67 [0.13 -3.47]	−0.56	.519	0.57 [0.10-3.14]
	Elderly	−0.15	.782	0.86 [0.29-2.55]	not included ^b^			not included ^b^		
Gender	Male	0.48	< .001	1.62 [1.24-2.11]	0.70	< .001	2.01 [1.47 - 2.76]	0.53	.003	1.70 [1.21-2.40]
Refugee Boat No.	1	0.94	< .001	2.56 [1.56-4.21]				0.87	.022	2.39 [1.14-5.01]
	2	1.17	.001	3.23 [1.60-6.51]				1.19	.003	3.27 [1.50-7.14]
	3	1.75	< .001	5.77 [3.58-9.29]				1.88	< .001	6.56 [3.71-11.58]
	4	−0.05	.863	0.95 [0.54-1.68]				−0.02	.956	0.98 [0.47-2.06]
	5	1.20	< .001	3.33 [1.73-6.42]				1.33	< .001	3.79 [1.82-7.90]
	6	1.80	< .001	6.05 [3.20-11.44]				1.84	< .001	6.30 [3.06-12.95]
	7	0.22	.651	1.24 [0.49-3.16]				0.41	.418	1.51 [0.56-4.09]
	8	1.79	< .001	5.99 [3.60-9.96]				1.98	< .001	7.25 [3.90-13.49]
	9	3.12	< .001	22.59 [12.70-40.19]				2.80	< .001	16.52 [8.16-33.44]
	10	not calculated^b^						not included ^b^		

Note: ^a^Subcategory is not calculated due to its small sample size

^b^Subcategory is not included because it is explained by the other subcategories

In addition to the envisaged subgroups, the analyses were carried out with subsets of the overall patient population: restricting the examinations to the subgroup of male adults yielded no crucial information other than the results already shown above. The same applies if patients with conditions normally not affecting vital signs (e.g. dermatological problems) are excluded or if refugee boats with incomplete data sets are excepted from the analysis.

### The patient population of people in distress at Sea

A summarised overview of the conditions/injuries noted is given in Table 4. Of the 448 patients treated, 83.9 % were male. At 18.3 % the proportion of males requiring treatment was significantly higher (*p* < .001) than the proportion of sick females (12.1 %). Only 5.6 % of the infants rescued required treatment. Of the 274 children, 14.2 % were sick. In the “adults” and “elderly” age groups, 17.1 % respectively 46.2 % respectively received treatment (*p* = .012).

**Table 4:** Analysis of demographic data, condition/injury patterns, and measures taken of the patient’s population

	Overall patient population
Number of sick/injured patients	*n* = 448
Age group	
▪ Infant	1/448 (0.2 %)
▪ Child	38/448 (8.5 %)
▪ Adult	403/448 (90.0 %)
▪ Elderly	6/448 (1.3 %)
Classification of symptoms	
▪ Dermatological problems	248/448 (55.4 %)
▪ Cardiovascular problems	99/448 (22.1 %)
▪ Pulmonary problems	20/448 (4.5 %)
▪ Abdominal problems/GI infection	5/448 (1.1 %)
▪ Orthopaedic problems	20/448 (4.5 %)
▪ Injuries/Traumatological problems	34/448 (7.6 %)
▪ ENT/OMS problems	8/448 (1.8 %)
▪ Ophthalmological problems	8/448 (1.8 %)
▪ Gynaecological problems	5/448 (1.1 %)
Initial treatment	
▪ Infusion therapy	35/448 (7.8 %)
▪ Analgesia	39/448 (8.7 %)
▪ Antibiotic treatment	10/448 (2.2 %)
▪ Bandages/wound cleaning	19/448 (4.2 %)
▪ Other medical measures	14/448 (3.1 %)
▪ Admission to emergency field hospital	62/365 (17.0 %)

Notes: The symptoms of 23 of the patients were not classified. The symptoms of 22 of the sick patients were assigned to two areas. Three pregnant women were also classified as sick/injured. Admission to emergency field hospital is not recorded in refugee boat no. 1 and 2

**Fig. 3:**
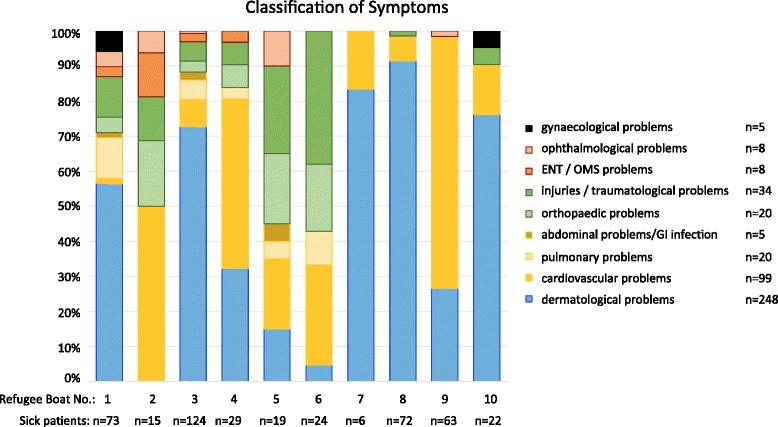
Overview and classification of symptoms of 448 rescued people from distress at sea. The stacked bar chart shows the proportion of main symptoms in refugee boat 1 to 10 as well as the absolute figures

55.4 % of the patients had skin conditions. Cardiovascular, lung and non-traumatic abdominal conditions had a proportion of 27.7 % together. The proportion of acute traumas, including orthopaedic symptoms, was 12.1 % and varied significantly between the individual refugee boats (cf. Fig. 3).

### Treatment during initial care

A total of 117 medical measures were taken during the initial care phase (cf. Table 4). 3.1 % of all rescued people were admitted to the emergency field hospital for further examination and/or treatment.

### Follow-on care

Both the people rated as healthy and those rated as in need of treatment were handed over to the Italian authorities as planned, without any noticeable deterioration in their condition. None of the rescued people went to the medical team with new symptoms during transportation, and none of them had to be evacuated prematurely. All rescued people could be handed over to the Italian authorities, respectively to a treatment facility on the Italian homeland within 24 to 36 h.

## Discussion

One aim of this secondary data analysis was to evaluate the condition/injury pattern of refugees in distress in the Mediterranean. Another was to verify whether pulse rate, SpO_2_ and CBT substantiated the need for initial assessment in this population. In this respect, the study submitted closes a gap because no investigation has been carried out to date on the initial assessment and initial care of refugees in distress in the Mediterranean, despite the rising number of refugees.

### Discussion on the initial assessment

By definition, the initial assessment performed here is not equivalent to the triage process carried out in disasters [6] or to the initial assessment of patients in Accident and Emergency (A&E) Departments [14].

The authors of pertinent works of reference discuss a variety of procedures that can be used in mass casualty situations [15–19]. Also there exist various initial assessment tools used to establish priorities in A&E departments not only for traumas, but also for mixed patient populations [14, 20–22]. The authors of this article believe that neither of these protocols is suitable for the situation described here because the present scenario, a very high proportion of people who were not in need of treatment are expected [19].

Another particular point is that the people rescued could have infectious conditions which made non-verbal communication almost entirely impossible due to the PPE. To make matters worse, only a very limited amount of time (<60 s) was allowed per rescued person for the triage process. In the light of the space available and the continuous influx of these people that prevailed during the rescue operations, it was essential to avoid a backlog building up. A backlog would have inevitably jeopardised the rescue of further people in distress at sea and endangered their lives. Houston et al. were able to show that even under normal conditions in an A&E Department, the median waiting time for traditional initial triage is 11 min and, at peak times, can be up to 105 min [23]. Ellebrecht et al. showed that triage in a mass casualty exercise even took an average of 53 s per patient [24]. Thus the initial assessment rate of 100 % and the collection rate of 72.1 % for additionally vital parameters are to be classified as good.

The idea of recording objectifiable parameters in order to triage the need for treatment, must nonetheless be rated as a failure. Relying on these parameters would have made it impossible to identify those who were sick or those who needed admission to the emergency field hospital (cf. sensitivity and specifity in Table 3).

In contrast to the findings of Ljunggren et al. no clinically useful model could be established between the state of health, the need for treatment, and admission to an emergency field hospital for any of the examined vital parameters [20] (cf. Fig. 2a-c for the uncategorised vital signs respectively Tables 2 and 3 for the categorised vital signs).

The authors’ opinion is that having the initial assessment carried out by an experienced emergency physician is a clear benefit. They account for this conclusion, which is in keeping with an overview by Abdulwahid et al. [25] the following facts:

- ▪ Certain relevant parameters were not recorded. These parameters included “respiratory rate” (not recorded due to lack of time) and “capillary reperfusion” (not recorded due to unfavourable light conditions) [15–18, 20].
- ▪ In addition, stress, physical exhaustion, and the climatic conditions led to pathologically higher PR although initial treatment had not been indicated [26].
- ▪ The typical population observed in an A&E Department is “sick/injured” per definition [20]. In our unselected collective of people rescued from distress at sea 83.1 % were healthy participants.

### Discussion on conditions and initial care

Neither the diagnostic tests carried out nor the initial care provided on board the frigate are comparable with established European standards [2] and medical care has to be provided for a maximum of 24 to 36 h only. This means that all data on incidence and classification in Table 4 and Fig. 3 must be interpreted as “provisional” data, meaning that the diagnoses are suspected diagnoses with the focus being on an urgent need for treatment. The refugees’ mental problems, as described in multiple overviews, were not recorded [5, 27, 28]. Acute self-endangerment or endangerment of others was not observed, however.

A large share of those rescued showed signs of physical exhaustion, resulting from the long ordeal of their journeys and factors such as malnutrition, lack of fluids [29]. Most of the people rescued were helped simply by being given fluids and food orally. Some, however, were so exhausted that parenteral fluid substitution was necessary (cf. Table 4). Other common conditions included dermatosis, scabies, conjunctivitis, respiratory tract conditions, and fever. Surgical symptoms included oropharyngeal abscesses, some of which had to be cleared surgically, old superinfected wounds, which were debrided and cleaned, or fresh lacerations resulting from knocks to the head, which were stitched.

Interestingly the pattern of symptoms differed significantly between the ten refugee boats: Whereas refugee boat no. 1 had a proportionate distribution basis like the overall patient collective, dermatological conditions dominated in refugee boats nos. 7, 8 and 10. Two other refugee boats (nos. 5 and 6) impressed with nearly 50 % of refugees with injuries and orthopaedic problems (cf. Fig. 3). It is unlikely that this distribution is due to special circumstances during the refugee’s time on sea. These times varied only between circa four and 18 h. We postulate that it is more likely that the different circumstances and the varying time (few days up to several weeks) before their trip over the Mediterranean Sea might be more influencing. Especially no violence has been observed or reported on board of the refugees’ boats.

However, in keeping with an analysis concerning Syrian and Palestinian refugees, we detected certain similarities in the condition pattern as well as a relevant proportion of untreated pre-existing problems and injuries [5, 30].

### Transferability of results

The population examined is in many respects consistent with international overviews with regard to demographic data [5, 27] and condition/injury patterns. Since there is currently no study on the specific problems associated with rescue operations and medical care in the Mediterranean however, no comparison can be drawn between the treatments initiated.

### Limitations

The results of the study presented here were obtained from routine data by means of a secondary data analysis. The sole purpose of this documentation was to carry out an initial assessment of all those rescued and to identify people who required further examination or treatment.

More information could have been acquired if it had been possible to archive the triage tags used for documentation. However, they were submitted to the Italian authorities for information purposes. Further studies should include the option of performing a comparison of preliminary on board diagnosis with the results of the regular in hospital care on the mainland. Hence the results of this secondary data analyses about refugees only confirmed that the medical infrastructure/personnel staffing on board of the frigate was suitable for this type of rescue mission.

### Conclusion

This study delivers important results on the initial assessment and initial treatment of people in distress at sea who were rescued by a German Navy frigate in support of the European Union EUNAVOR-MED mission. Despite the time pressure and limited communication, the overall impression gained by an experienced emergency physician was a valid decision criterion that ensured that no patient was overlooked. The additional vital parameters collected (PR, CBT and SpO_2_) were not suitable for distinguishing between sick and healthy people rescued from distress at sea.

The condition and injury spectrum consisted to a large extent of dermatological conditions that can be accounted for by the hygiene conditions that prevailed during the refugees’ flight. Lack of fluid and pre-existing conditions were also common. Traumata requiring treatment, including new and old injuries resulting from acts of violence and accidents, are also likely to be found. The medical team must be prepared to treat localised and systemic infectious conditions and stringently apply appropriate hygiene measures.

## Additional file

Additional file 1: Excerpt of the underlying study variables with explanations and degree. (DOCX 100 kb)

## Abbreviations

95 % CI: 95 % confidence interval
A&E: accident and emergency (department)
CBT: core body temperature [°C]
ENT: ear, nose, throat
GI: Gastrointestinal Tract
OMS: oral and maxillofacial surgery
OR: odds ratio
PPE: personnel protective equipment
PR: pulse rate [/min]
R: regression coefficient
SpO_2_: peripheral oxygen saturation [%]

## Footnotes

1 Note: The Federal German Medical Association doesn’t offer the speciality “Emergency Physician” in their Specialty Training Regulations. So the German Armed Forces (Bundeswehr) is training their physician anaesthesiologists in dealing with emergencies on mission. Nearly all anaesthesiologists are trained in ATLS©, ETC., ALS etc. They do not only work in the operation theatre, but also on Intensive Care Units as well as in the prehospital setting (EMS and HEMS). Most of them have special certificates in “Intensive Care Medicine” and “Prehospital Emergency Medicine”. Additionally, the A&E Departments of the four German Armed Forces Hospitals are led by anaesthesiologists and nearly all consultants do regular shifts in A&E Departments.
