# Supplementary material for: Initial assessment and treatment of refugees in the Mediterranean Sea (a secondary data analysis concerning the initial assessment and treatment of 2656 refugees rescued from distress at sea in support of the EUNAVFOR MED relief mission of the EU)
Source: Scand J Trauma Resusc Emerg Med. 2016 May 20;24:75. doi: 10.1186/s13049-016-0270-z (PMC4873997; doi:10.1186/s13049-016-0270-z)
Supplement: Additional file 1: — Excerpt of the underlying study variables with explanations and degree. (DOCX 100 kb) [file 13049_2016_270_MOESM1_ESM.docx]

## Electronic Supplement 1

Excerpt of the underlying study variables with explanations and degree.

| **Variable** | **Description** | **Degree** | |
| --- | --- | --- | --- |
| Study ID | Study ID (unambiguous serial number) |  | |
| Boat ID | Unambiguous serial number of the refugee boat in distress at sea | 1,2,3,…. | |
| Date / time | Time of initial contact, pseudonymised by means of consecutive letters. An additional index number is specified if several refugee boats are recovered without a stop at a port | A, B, Gl, C2, C3, D... | |
| Time on see | Estimated time on see of the refugee boat | hours | |
| Gender | Gender of rescued person / refugee in distress at sea | m= male f = female | |
| Age group | Coding of estimated age of rescued person / refugee in distress at sea in four age groups | 1 = infant 2 = child (young child up to and including puberty)  3 = adult  4 = elderly (presumed >60a) | |
| **Vital Signs / initial assessment** | | | |
| - Pulse rate (PR) | Peripheral pulse rate  Classified in categories:   - ≤ 39 [/min] - 40-49 [/min] - 50-59 [/min] - 60-80 [/min] - 81-120 [/min] - 121-150 [/min] - ≥ 151 [/min] | | Raw value [/min]  yes / no  yes / no  yes / no  yes / no  yes / no  yes / no  yes / no |
| - Oxygen saturation (SpO_2_) | Pulsoxymetrically determined peripheral oxygen saturation  Classified in categories:   - ≤ 84 [%] - 85-89 [%] - 90-95 [%] - 96-100 [%] | | Raw value [%]  yes / no  yes / no  yes / no  yes / no |
| - Core body temperature [CBT] | Core body temperature (measured auricularly)  Classified in categories:   - ≤ 34.9 [°C] - 35.0-35.9 [°C] - 36.0-37.5 [°C] - 37.6-39.0 [°C] - ≥ 39.1 [/min] | | Raw value [°C]  yes / no  yes / no  yes / no  yes / no  yes / no |
| Sick | Rescued person / refugee in distress at sea diagnosed as sick / injured while on board. In this case, the term 'sick' is used to mean the same as "injured". | | yes / no |
| **Classification of symptoms** | | | |
| - Dermatological problems | Skin conditions / symptoms | yes / no | |
| - Cardiovascular problems | Conditions / symptoms from the cardiovascular sphere, including all consequences of dehydration and seasickness | yes / no | |
| - Pulmonary problems | Lung / respiratory tract conditions / symptoms | yes / no | |
| - Abdominal problems / Gastrointestinal Tract (GI) infection | Abdominal / gastroenterological conditions / symptoms (excluding consequences of violence) | yes/no | |
| - Orthopaedic problems | Orthopaedic problems | yes / no | |
| - Injuries / Traumatological problems | all injuries resulting from external action | yes / no | |
| - ENT / OMS problems | Head / neck conditions / injuries / symptoms | yes / no | |
| - Ophthalmological problems | Eye conditions / symptoms / injuries | yes / no | |
| - Gynaecological problems | Gynaecological conditions / symptoms | yes / no | |
| Pregnancy | Pregnancy specified (not recorded for boats 1 and 2) | yes / no | |
| Condition | Description of condition / injury | Free text | |
| **Initial treatment** | | | |
| - Infusion therapy | Intravenous infusion therapy | yes / no | |
| - Analgesia | Administration of analgesics | yes / no | |
| - Antibiotic treatment | Anti-infective therapy | yes / no | |
| - Bandages / wound cleansing | Replacement of bandages / splinting | yes / no | |
| - Other medical measures | All other medical measures | yes / no | |
| Emergency field hospital | Treatment at the emergency field hospital (not recorded for boats 1 and 2) | yes / no | |
